# Determinants of anemia severity levels among children aged 6–59 months in Ethiopia: Multilevel Bayesian statistical approach

**DOI:** 10.1038/s41598-022-20381-7

**Published:** 2023-03-13

**Authors:** Tesfa Sewunet Alamneh, Alemakef Wagnew Melesse, Kassahun Alemu Gelaye

**Affiliations:** Department of Epidemiology and Biostatistics, Institute of Public Health, College of Medicine and Health Sciences, University of Gondar, Gondar, Ethiopia

**Keywords:** Medical research, Risk factors

## Abstract

Anemia is a widespread public health problem that affects all stages of life particularly preschool children and pregnant mothers. Anemia among children had significant impact on their growth, development, school performance and mortality. Different strategies like deworming of young children, vitamin A supplementation for children aged 6–59 months, and ferrous sulphate supplementation and provision of insecticide treated bed net for pregnant women were designed to control and prevent anemia. Also, previous studies on anemia factors were conducted but they were not considering the ordered nature of anemia. Therefore, this study aimed to identify the factors of anemia severity levels among children aged 6–59 months in Ethiopia by using ordinal analysis based on Bayesian hierarchical statistical approach**.** A secondary data analysis was conducted using the 2016 Ethiopian Demographic and Health Survey data. A total of 8483 weighted children were included. Due to the ordered nature of the anemia and nested nature of DHS data, ordinal logistic regression model based on hierarchical Bayesian statistical approach was employed to identify the determinants of anemia severity levels. In this study, moderate anemia level was found to be the commonest type which accounts around 29.4%. Female children, poorer, middle, and richest wealth index, primary maternal education and having ANC visit had lower risk of having higher order of anemia. Moderate maternal anemia and stunted children had higher chance of having higher order of anemia. Children age had significant different effect on mild and moderate anemia. Meanwhile, multiple birth/s and deworming had effect on moderate anemia. In addition, normal birth weight had also significant and different effect on mild and severe anemia and history of feverlike illness on mild anemia. The prevalence of anemia among children aged 6–59 months anemia was found to be a severe public health problem. Children age, sex, maternal education, child stunting, history of fever, multiple birth, birth weight, provision of deworming and maternal anemia was found to be the most important factors for child anemia severity levels. Therefore, intervention efforts to control and prevent anemia in Ethiopia requires targeting of these hindering factors.

## Introduction

Anemia is the condition in which the oxygen carrying capacity or the hemoglobin (Hgb) concentration of Red Blood Cell count (RBC) are lower than normal level of healthy individual and becoming insufficient to meet physiological needs^1^. It is one of the most common and widespread public health problem in the world that affects all stages of life particularly preschool children and pregnant mothers^2^. According to world health organization (WHO), Anemia becomes a public health problem when it affects 5% or more of the population. It is classified as mild, moderate, and severe public health problem when it affects 5–19.9, 20–39.9 and greater than 40% of the population respectively. Based on the Hgb value, It has also different severity levels(i.e. none, mild, moderate and sever)^3^.

Globally, anemia affects 1.62 billion people and nearly half (47.4%) of those population were pre-school children. Approximately two-thirds of under five children live in Africa and south East Asia were anemic^4^. Whereas in sub-Saharan African countries, 62.3% of under five children were affected by anemia^5^. It is also one of the common Nutritional problems in Ethiopia which affects around 57% of children aged from 6 to 59 months^6^. Although anemia is treatable and preventable health condition, it has negative impact on child growth, language development, school performance and child mortality^7–9^.

According to different studies conducted in different areas, anemia was affected by socio-demographic and economic factors such as sex^10^ and age of child^11^, residence^12^, mother’s age^12^, maternal education^13^ and employment^14^. It was also affected by heath related conditions such as feverile illness like malaria^15^, diarrheal disease^16^, birth weight^17^ and maternal anemia^18^. There were also different strategies designed to prevent child hood anemia like deworming of children aged from 6 to 59 months^19^, vitamin A (Vit A) supplementation for children aged 6–59 months^20^, and ferrous sulphate supplementation and provision of insecticide treated bed net for pregnant mother to prevent maternal anemia which in turn affects anemia of children^21^.

Even though different strategies were implemented to prevent and control anemia, it remains a major public health problem in the country. Previous Research conducted on the magnitudes and factors of anemia were conducted in specific areas or groups which were not representative of the entire population^11,14,16,22–25^. These studies also were not considering the ordered nature of anemia (i.e., none, mild, moderate, and severe) but they simply considered it as yes or no type of data nature. But the effect of those factors on mild, moderate, and severe anemia might not the same. So, considering anemia as a binary data may result in loss of information. Moreover, limited studies were available based on Bayesian statistical approach which is a good approach for studies in medical science, hierarchical data like EDHS and it gives reliable and consistent estimate than classical approach by using the prior information in addition to the data^26^. Hence, identifying the determinants of anemia severity by using nationally representative data have a paramount importance to design effective intervention strategies and implementing effective measures for controlling and preventing anemia.

Therefore, the present study aimed to identify the determinants of anemia levels among children aged 6–59 months in Ethiopia by using ordinal analysis based on Bayesian hierarchical statistical approach.

## Methods

### Study setting

This study was conducted in Ethiopia which is an East African country with an estimated population of 115.5 million that makes it second most populous country in Africa^27^. It has a high central plateau that varies from 1290 to 3000 m (4232–9843 ft) above sea level, with the highest mountain reaching 4533 m (14,872 ft)^28^. In Ethiopia, 75% of the land are malaria’s areas and more than 54 million people are vulnerable for malaria infection^29^. Moreover, the prevalence of intestinal parasite infections was high in Ethiopia which affects 48% in preschool and school-age children^30^. Administratively, Ethiopia is federally decentralized in to 9 regions and two city administrations and regions are divided into zones, and zones, into administrative units called districts. Each district is further subdivided into the lowest administrative unit, called kebele. Regarding to the health care system in Ethiopia, the fourth health sector development plan introduced a three-tier health-service delivery system. This system was arranged by including Primary health care unities (i.e., health posts and health centers) and primary hospitals at primary level, general hospitals at secondary level, and specialized hospitals at tertiary level^31^.

### Data source, study population and sampling technique

This study was based on the EDHS 2016 data which was a nationally representative sample conducted from January 18 to June 27, 2016. Regarding the sampling technique, two stage stratified cluster sampling technique were employed to select study participants. Stratification was conducted by separating each region into urban and rural areas. In the first stage, 645 enumeration areas (202 from urban area) were selected with probability proportional to the enumeration area size and with independent selection in each sampling stratum.

In the second stage, 24–32 households from each cluster were selected with an equal probability systematic selection from the household listing.

For this study, the data was accessed from the Measure demographic and health survey (DHS) website (http://www.measuredhs.com). The study population were children aged from 6 to 59 months who had born 5 years prior to 2016 DHS study in Ethiopia and in the selected enumeration areas.

### Study variables

In EDHS anaemia testing was conducted for all children from aged 6 to 59 months for whom consent was obtained from their parents or other adults responsible for them. Blood sample was drawn from a drop of blood taken from a finger prick (or a heel prick in children aged from 6 to 11 months) and collected in a microcuvette. Haemoglobin analysis was carried out on-site using a battery-operated portable HemoCue analyser.

The response variable for this study was anemia level among children aged from 6 to 59 months which has four levels^31^:

$$\begin{aligned} {\text{i}}.{\text{e}}.~~~~~~~~~~~~~~~~~ & {\text{none}}:{\text{ if Hgb value}}~{\text{11 mg}}/{\text{dl}} \\ & {\text{mild}}:{\text{ if Hgb value ranges from 1}}0.0 \, {\text{to}}\, 10.{\text{9 mg}}/{\text{dl}} \\ & {\text{moderate}}:{\text{ if Hgb value ranges from 7}}.0\, {\text{to}}\,{\text{9}}.{\text{9 g}}/{\text{dl}}),{\text{ and}} \\ & {\text{severe}}:~{\text{if Hgb value less than 7}}.0{\text{ mg}}/{\text{dl}}) \\ \end{aligned}$$

In this study, both individual and community-level explanatory variables were considered. The individual-level variables included were sex, age of child, age of mother, wealth index, mass media exposure, educational status, marital status, maternal anemia, parity, ANC visit, place of delivery, birth spacing, multiple birth, birth weight, recent history of diarrhea and fever, stunting, Vit.A supplementation, drugs for intestinal parasites, immunization. The Place of residence and CDI were considered as community-level factors. The community-level variable, CDI, was measured based on the availability of three basic services in the community: improved water supply, electricity city, and improved sanitation services. It is classified as:

- Low: community with none of those three services
- Medium: community with one or two services
- High: community with three services^32^.

### Data management and statistical analysis

After accessing the data from measure DHS, the variables of the study were extracted from Birth recorded data set of EDHS data using STATA version 14. The data was weighted using sampling weight during any statistical analysis to adjust for unequal probability of selection due to the sampling design used in EDHS data. Hence, the representativeness of the survey results was ensured.

A two-level multivariable ordinal logistic regression analysis was used to estimate the effect of explanatory variables on anemia severity. The data has two levels with a group of J EAs and within-group j (j = 1, 2…, J), a random sample nj of level-one units (individual children). The response variable is denoted by ${\text{Y}}_{{{\text{ij}}}} = 0{\text{ if the }}i{\text{th children are in the }}j{\text{th EAs had not anemia in the test result}}$

1. if ith children are in the jth EA’s had mild anemia test result
2. if ith children are in the jth EA’s had moderate anemia test result
3. if ith children are in the jth EA’s had severe anemia test result

So, appropriate inferences and conclusions from this data does require advanced modeling techniques like multilevel modeling, which contain variables measured at different levels of the hierarchy, to account the nested effect^33^. Four models were fitted for the data. The first model was an empty model without any explanatory variables, to calculate the extent of cluster variation on anemia level. Variation between cluster (EAs) were assessed by computing Intra-class Correlation Coefficient (ICC), Proportional Change in Variance (PCV) and Median Odds Ratio (MOR). The ICC is the proportion of variance explained by the grouping structure in the population. It was computed as: ICC = $\frac{{\sigma _{\mu } ^{2} }}{{\sigma _{\mu } ^{2} + \pi ^{2} /3}}$; where: the standard logit distribution has variance of $\pi ^{2} /3$, ${{\sigma }_{\mu }}^{2}$ indicates the cluster variance. Whereas PCV measures the total variation attributed by individual level and community level factors in the multilevel model as compared to the null model. It was computed as: $\frac{\mathrm{variance of null model}-\mathrm{variance of full model}}{\mathrm{variance of null model}}$^34^. MOR is defined as the median value of the odds ratio between the cluster at high risk and cluster at lower risk of higher anemia level when randomly picking out two clusters (EAs). It was computed as **:**MOR = exp ($\sqrt{2*{{\upsigma }_{\upmu }}^{2}*0.6745}$) $\sim$ MOR = exp ($0.95*{\upsigma }_{\upmu }$)^35^. The second model was adjusted with individual level variables; the third model was adjusted for community level variables while the fourth was fitted with both individual and community level variables.

The analysis was conducted based on Bayesian statistical approach which assumes parameters are unknown and random that follows a certain probability distribution. This approach had three components which are the likelihood function, prior distribution, and posterior distribution^36^.

### Likelihood function

It is the key component of Bayesian statistical approach that reflects information about the parameters contained in the data. But the data used for this analysis had ordered response with two hierarchies which requires to employ multilevel ordinal analysis. For ordered data, there are different types of ordinal models. This study first considers the common and most applicable model for ordinal response variable which is cumulative logit model. It is an extension of binary logistic regression model and estimate the odds of being beyond a particular level of the response^37^. It is formulated as

$$\mathrm{logit}\left({\mathrm{p}(\mathrm{y}>}_{{k}_{ij}}\right)=\mathrm{log}\left(\frac{\mathrm{p}\left({\mathrm{y}}_{\mathrm{ij}}>\mathrm{k}\right)}{\mathrm{p}\left({\mathrm{y}}_{\mathrm{ij}}\le \mathrm{k}\right)}={\mathrm{a}}_{\mathrm{k}}+{\mathrm{B}}_{0}+\sum_{\mathrm{h}=1}^{\mathrm{l}}{\mathrm{B}}_{\mathrm{h}}{\mathrm{X}}_{\mathrm{hij}}+{\upgamma }_{0\mathrm{j}}\right.$$

where k = 1, 2, K − 1; ${\mathrm{a}}_{\mathrm{k}}$ is cut point of response variable; ${\mathrm{B}}_{\mathrm{p}}$ are fixed coefficients of the corresponding predictor variables and ${\upgamma }_{0\mathrm{j}}$ random intercept coefficient. A positive logit coefficient indicates that an individual is more likely to be in a higher category as opposed to a lower category of the outcome variable^33^. But this model has constrained Proportional Odds Assumption (POA) or parallel lines assumption. That is, for each cumulative logit the parameters of the models are the same, except for the intercept^38^. This assumption was tested by using brant test of parallel lines and it becomes significant for overall model as well as some variable (i.e., POA assumption was not satisfied). Then Continuation ratio and Adjacent-categories logistic regression model with category specific effect were fitted to relax the POA.

### Continuation ratio model

It also called sequential model which compares the probability of a response higher level equal (${\mathrm{y}}_{\mathrm{ij}}>\mathrm{k})$ to a given category (Y = k)^39^.

The model is given as

$$\mathrm{logit}\left(\frac{\mathrm{p}\left({\mathrm{y}}_{\mathrm{ij}}>\mathrm{k}\right)}{\mathrm{p}\left({\mathrm{y}}_{\mathrm{ij}}=\mathrm{k}\right)}=\mathrm{log}\left(\frac{\mathrm{p}\left({\mathrm{y}}_{\mathrm{ij}}>\mathrm{k}\right)}{\mathrm{p}\left({\mathrm{y}}_{\mathrm{ij}}=\mathrm{k}\right)}={\mathrm{a}}_{\mathrm{k}}+{\mathrm{B}}_{0}+\sum_{\mathrm{h}=1}^{\mathrm{l}}{\mathrm{B}}_{\mathrm{h}}{\mathrm{X}}_{\mathrm{hij}}+{\upgamma }_{0\mathrm{j}}\right.\right.$$

where k = 1, 2, K − 1; ${\mathrm{a}}_{\mathrm{k}}$ is cut point of response variable; ${\mathrm{B}}_{\mathrm{p}}$ are fixed coefficients of the corresponding predictor variables and ${\upgamma }_{0\mathrm{j}}$ random intercept coefficient.

### Adjacent-categories model

It is the modeling of pairs of adjacent categories of the ordinal response variable. This model compares the probability of being in the higher category relative to the lower category. The model can be expressed as

$$\mathrm{logit}(\mathrm{p}\left({\mathrm{y}}_{\mathrm{ij}}=\mathrm{k}+1\right))=\mathrm{log}\left(\frac{\mathrm{p}\left({\mathrm{y}}_{\mathrm{ij}}=\mathrm{k}+1\right)}{\mathrm{p}\left({\mathrm{y}}_{\mathrm{ij}}=\mathrm{k}\right)}\right)={\mathrm{a}}_{\mathrm{k}}+{\mathrm{B}}_{0}+\sum_{\mathrm{h}=1}^{\mathrm{l}}{\mathrm{B}}_{\mathrm{h}}{\mathrm{X}}_{\mathrm{hij}}+{\upgamma }_{0\mathrm{j}}$$

where k = 1, 2, K − 1; ${\mathrm{a}}_{\mathrm{k}}$ is cut point of response variable; ${\mathrm{B}}_{\mathrm{p}}$ are fixed coefficients of the corresponding predictor variables and ${\upgamma }_{0\mathrm{j}}$ random intercept coefficient^38^.

### Prior distribution

It is the probability distribution that represents the prior information associated with the parameters of interest. In Bayesian two common types of priors were used (Informative and Non-informative priors). An informative prior is a prior distribution that is used when information about the parameter of interest is available before the data is collected. It can obtain from previous studies, expert knowledge (experience) and a combination of both. Due to lack of these sources, non-informative (flat) prior distributions, which gives less value to the data collected before while giving high attention to the data or likelihood, were used for this study. Normal flat prior distribution for the population level parameters, and uniform prior distribution for the group level parameters were used.

### Posterior distribution

It represents the total knowledge about the parameters after the data have been observed. It is obtained by multiplying the prior distribution over all parameters by the likelihood function (i.e., $f\left(\theta |y\right)\propto \mathrm{f}\left(\mathrm{y}|\uptheta \right)*\mathrm{f}(\uptheta )$). Where $\mathrm{f}(\uptheta )$ is the prior distribution; $\mathrm{f}\left(\mathrm{y}|\uptheta \right)$ is the likelihood of the data and $f\left(\theta |y\right)$ is posterior distribution^40^.

Simulation technique was applied by using Bayesian regression model using stan (BRMS) package in R^41^ with two chains that have 8000 with 3000 warm up iterations. The parameters were allowed to be initiated randomly in the simulation procedure. The samples were drawn by using one variant of Markov chain Monte Carlo (MCMC) algorithm called No-U Turn Sampler (NUTS) which improves the limitations of Hamiltonian monte Carlo’s (HMC) by introducing the slice variable that sampled uniform distribution in the sampling procedure. Like HMC, NUTS sampler surpasses the random walk behavior of Gibbs and Metropolis- Hasting sampler by including the clever auxiliary variable. This property of HMC and NUTS sampler make more efficient than other MCMC sampling techniques with small iteration^42^.

The results from a given distribution are not deemed reliable until the chain has reached its stationary assumption. But the inference becomes appropriate when target distributions is well converged. Therefore, monitoring its convergence is essential for producing reliable results from the posterior distribution. The convergence of the targeted distribution was assessed by trace plot, density plot, effective sample size and R hat statistics.

Unlike classical approach, Akaike Information Criterion (AIC), Bayesian Information Criterion (BIC) and Deviance information criterion (DIC) are not appropriate model section criteria for Bayesian statistical approach. To overcome this, this study computes Leave-One-Out cross-validation (LOO) and the Watanabe Akaike Information Criterion (WAIC). Models were selected using LOO because WAIC, which is computed as log predictive density for each data point minus estimated effective number of parameters, becomes unreliable if any of estimated effective number parameter exceeds 0.4^43^. But in this case, it becomes in tens. Based on LOO results, which Estimates out-of-sample pointwise predictive accuracy using posterior simulations, the model with smaller LOOIC was selected as the best fitted model. Adjusted odds ratio (AOR) with 95% Credible Interval (CrI) from best fitted model was used to select variables which have significant association with anemia level among children aged 6–59 months.

### Ethical approval

This study is a secondary data analysis from the EDHS data, so it does not require ethical approval. For conducting this study, online registration and request for measure DHS were conducted. The dataset was downloaded from DHS on-line archive after getting approval to access the data.

### Patient and public involvement

In this study, patients and the public were not involved in the study design or planning of the study. Furthermore, since we used secondary analysis DHS data patients were not consulted to interpret the results and were not invited to contribute to the writing or editing of this document for readability or accuracy.

## Results

### Background characteristics

A total of 8483 weighted children aged from 6 to 59 months were included in this study. Among thus, the median age was 31 with inter quartile range of (18,46) months. Majority of the study participants (89.85%) were rural dwellers. Regarding to child mother characteristics, the majority (72.23%) of the mother were aged between 20 and 35 years, 67.02% of them had no formal education, 93.88% were married, 57.84% had no ANC visit and 75.01 of them were deliver their baby at home. Moreover, nearly half (44.29%) of children aged from 6 to 59 months were supplemented with Vit A, 12.86% were dewormed, 35.44% were stunted and 15.20% had recent history of fever (Table 1).

**Table 1:** Background characteristics of study participants.

Characteristics	Frequency (n = 8483)	Percent (%)
**Child sex**		
Male	4398	49.93
Female	4085	48.15
**Residence**		
Rural	861	10.15
Urban	7622	89.85
**Mother’s age**		
< 20	226	2.67
20–35	6127	72.23
> 35	2130	25.11
**Marital status**		
Not married	519	6.12
Married	7964	93.88
**Educational status**		
No education	5685	67.02
Primary	2282	26.89
Secondary +	517	6.09
**Maternal employment**		
Not employed	4626	54.53
Employed	3857	45.47
**Wealth index**		
Poorest	1989	23.44
Poorer	1990	23.45
Middle	1824	21.49
Richer	1540	18.15
Richest	1142	13.46
**MM exposure**		
No	7025	82.81
Yes	1458	17.19
**CDI**		
Low	3512	41.40
Moderate	4584	54.04
High	387	4.56
**Parity**		
< 5	4732	55.78
≥ 5	3751	44.22
**ANC visit**		
Yes	3577	42.16
No	4906	57.84
**Place of delivery**		
Home	6363	75.01
Health facility	2120	24.99
**Birth spacing (year)**		
≥ 2	5492	64.75
< 2	2991	35.25
**Maternal anemia**		
None	5959	70.25
Mild	1876	22.11
Moderate	522	6.15
Severe	128	1.50
**Multiple birth**		
Yes	209	2.45
No	8275	97.55
**Birth weight**		
LBW	2130	25.11
Normal	3612	42.58
Overweight	2741	32.31
**Diarrhea**		
Yes	1091	12.85
No	7393	87.15
**Fever**		
Yes	1290	15.20
No	7194	84.80
**Stunting**		
Yes	3006	35.44
No	5477	64.56
**Vit.A**		
Yes	3757	44.29
No	4726	55.71
**Drugs for IP**		
Yes	1092	12.86
No	7392	87.14
**Immunization**		
Complete	1560	18.39
Incomplete	6923	81.61

### Prevalence of anemia

**Figure 1:**
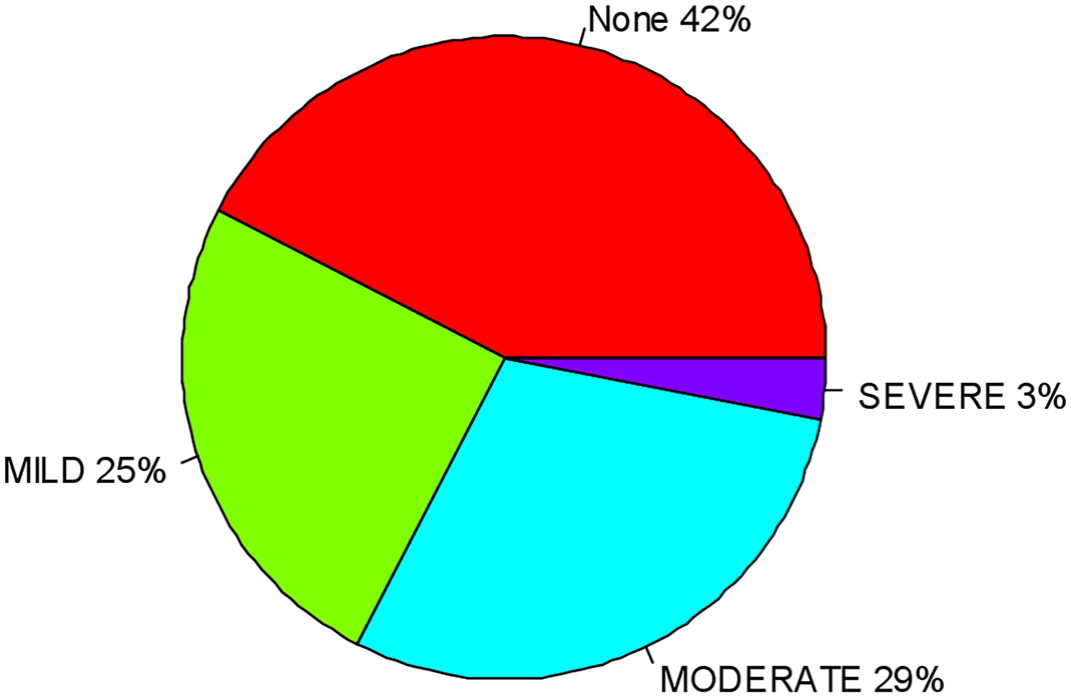
Prevalence of anemia among children aged from 6 to 59 months in Ethiopia, 2016.

The overall prevalence of anemia among children aged 6–59 months was 57.56% (95% CI 55.94, 59.16). Moderate anemia was the commonest (29.41%) type of anemia among children aged from6 to 59 months (Fig. 1).

### Determinants of anemia

We fitted four models based on hierarchical Bayesian statistical approach to identify the determinants of anemia level among children aged from 6 to 59 months. The convergence of these models to targeted distribution were assessed by viewing the mixture of two chains in trace plot, symmetry of its distribution in the density plot as well as the value of R hat close to one and adequacy of effective sample size. In this case the R hat value for all parameters were one, the effective sample size was adequate (> 1000), the chains were well mixed in the trace plot and exhibits symmetric distribution in the density plot.

In the first model, ICC indicated that 18.01% of the total variability for anemia level was due to differences between clusters/EA’s, with the remaining unexplained 81.99% was attributable to the individual differences. Regarding to PCV, about 50.58% of the variability in anemia level was explained by the full model. The median odds ratio also revealed that anemia among children aged from 6 to 59 months was heterogeneous among clusters. It was 2.24 in the null model which implies children within cluster of having higher risk for higher level of anemia had 2.24 times higher chance of having higher level of anemia as compared with children with in cluster of having lower risk if children aged 6 to 59 months were selected randomly from two different clusters (EAs).

Based on the information criterion, the full model (model 4) with adjacent category were selected as the best fitted model with smallest LOOIC value. In this model sex, child age, maternal education, wealth index, ANC visit, maternal anemia, multiple birth, birth weight, deworming, stunting and history of fever were the important factors for anemia level in children aged from 6 to 59 months.

According to this study, female children had 6% (AOR = 0.94, 95% CrI 0.88–0.98) lower risk of having higher order of anemia. Regarding to child age, it had different effects on the levels of anemia with a month increase of child age the likelihood of having mild anemia and moderate anemia were reduced by 3% (AOR = 0.97, 95% CrI 0.96–0.97) and 3% (AOR = 0.97, CrI 0.97, 0.98), respectively. Improvement in wealth index lowers the risk of having higher level of anemia; 16% (AOR = 0.84, 95% CrI 0.77–0.93), 22% (AOR = 0.78, 95% CrI 0.71–0.86), and 28% (AOR = 0.72, 95% CrI 0.62–0.84) of lower risk of having higher level of anemia for poorer, middle, and richest respectively. In addition, children from mothers having primary education had 9% (AOR = 0.91, 95% CrI 0.84–0.97) lower risk of having higher order of anemia as compared to children from mother having no education. Besides, children from mothers having ANC visit had 7% (AOR = 0.93, 95% CrI 0.86, 0.99) lower risk of having higher order of anemia.

This study revealed that the odds of having higher level of anemia were 1.22 (95% CrI 1.13–1.55) times for children from mother having moderate anemia as compared to children from nonanemic mother. Stunted children had 32% (AOR = 1.32, 95% CrI 1.24–1.40) higher chance of having higher order of anemia. But multiple birth, birth weight, history of feverile illness and deworming had different effect on levels of anemia. The result implied that children with normal birth weight had 16% (AOR = 0.84, 95% CrI 0.73–0.97) and 55% (AOR = 0.45, 95% CrI 0.34- 0.65) lower risk for mild and severe anemia respectively. While children with overweight had 61% (AOR = 0.39, 95% CrI 0.27–0.55) lower risk for severe anemia as compared to LBW. Like that of birth weight, history of febrile illness had different effects on each level of anemia. But its effect was significant for mild level of anemia with 34% (AOR = 1.34, 95% CrI 1.14–1.57) higher risk of having mild anemia. The likelihood of having moderate anemia were 2.89 (95% CrI 1.28–6.18) times for children who have multiple birth type. But multiple birth had no effect on mild and severe levels of anemia. Provision of drugs for deworming of children were significantly related with their anemia level. It lowers the risk of having moderate anemia by 21% (AOR = 0.79, 95% CrI 0.65–0.95) but its effect on mild and severe anemia levels were not significant (Table 2).

**Table 2:** Multivariable ordinal logistic regression based on hierarchical Bayesian statistical approach result of factors associated with anemia of children aged from 6 to 59 months in Ethiopia, EDHS 2016**.**

Characteristics	Model 1 (95% CrI)	Model 2 AOR (95% CrI)	Model 3 AOR (95% CrI)	Model 4 AOR (95% CrI)
**Maternal age**				
< 20		1		1
20–35		0.91 (0.76, 1.09)		0.92 (0.76, 1.10)
> 35		0.89 (0.74, 1.08)		0.89 (0.73, 1.10)
**Child sex**				
Male		1		1
Female		0.93 (0.88, 0.99)		0.94 (0.88, 0.98)*
**Residence**				
Rural			1	1
Urban			1.50 (1.14, 1.98)	0.96 (0.78, 1.19)
**Marital status**				
Not married		1		1
married		0.91 (0.81, 1.03)		0.92 (0.81, 1.03)
**Educational status**				
No education		1		1
Primary		0.91 (0.84, 0.98)		0.91 (0.84, 0.97)*
Secondary +		0.94 (0.81, 1.07)		0.93 (0.80, 1.08)
**Marital status**				
Not married		1		1
Married		0.91 (0.81, 1.03)		0.92 (0.81, 1.03)
**Employment**				
Not employed		1		1
Employed		0.92 (0.86, 0.99)		0.92 (0.87, 1.02)
**Wealth index**				
Poorest		1		1
Poorer		0.85 (0.78, 0.93)		0.84 (0.77, 0.93)*
Middle		0.79 (0.72, 0.87)		0.78 (0.71, 0.86)*
Richer		0.91 (0.82, 1.02)		0.90 (0.81, 1.01)
Richest		0.75 (0.65, 0.86)		0.72 (0.62, 0.84)*
**MM exposure**				
No		1		1
Yes		0.95 (0.87, 1.05)		0.95 (0.87, 1.05)
**CDI**				
Low			1	1
Moderate			1.07 (0.97, 1.19)	1.08 (0.99, 1.17)
High			0.87 (0.64, 1.18)	0.99 (0.81, 1.24)
**Parity**				
< 5		1		1
≥ 5		0.98 (0.92, 1.06)		0.98 (0.91, 1.05)
**ANC visit**				
No		1		1
Yes		0.93 (0.87, 0.99)		0.93 (0.86, 0.99)*
**Place of delivery**				
Home		1		1
Institution		0.95 (0.88, 1.04)		0.95 (0.87, 1.03)
**Birth space (year)**				
< 2		1		1
≥ 2		0.95 (0.89, 1.01)		0.95 (0.89, 1.01)
**Maternal anemia**				
None		1		1
Mild		1.01 (0.85, 1.41)		1.08 (0.83, 1.39)
Moderate		1.25 (1.08, 1.59)		1.22 (1.13, 1.55)*
Severe		0.93 (0.73, 1.18)		0.91 (0.71, 1.15)
**Diarrhea**				
No		1		1
Yes		0.93 (0.85, 1.02)		0.93 (0.85, 1.02)
**Stunting**				
No		1		1
Yes		1.32 (1.24, 1.41)		1.32 (1.24, 1.40)*
**Vit.A**				
No		1		1
Yes		0.98 (0.98, 1.04)		0.98 (0.92, 1.04)
**Immunization**				
Incomplete		1		1
Complete		0.97 (0.89, 1.05)		0.97 (0.89, 1.05)
**Child age**				
Mild		0.969 (0.96, 0.97)		0.97 (0.96, 0.97)*
Moderate		0.975 (0.97, 0.98)		0.97 (0.97, 0.98)*
Severe		0.98 (0.97, 0.99)		0.99 (0.98, 1.03)
**Multiple birth**				
No		1		
**Yes**				
Mild		1.11 (0.80, 1.55)		1.11 (0.79, 81.53)
Moderate		0.97 (0.89, 1.07)		1.04 (0.99, 1.11)
Severe		2.86 (1.26, 6.12)		2.89 (1.28, 6.18)*
**Birth weight**				
LBW		1		1
**Normal**				
Mild		0.85 (0.74, 0.98)		0.84 (0.73, 0.97)*
Moderate		1.15 (0.99, 1.34)		1.15 (0.98, 1.34)
Severe		0.49 (0.36, 0.66)		0.45 (0.34, 0.65)*
**Overweight**				
Mild		0.98 (0.84, 1.14)		0.98 (0.84, 1.14)
Moderate		1.05 (0.89, 1.23)		1.05 (0.89, 1.23)
Severe		0.39 (0.28, 0.55)		0.39 (0.27, 0.55)*
**Drug for IP**				
No		1		1
**Yes**				
Mild		1.05 (0.89, 1.45)		1.03 (0.91, 1.42)
Moderate		0.79 (0.66, 0.95)		0.79 (0.65, 0.95)*
Severe		1.13 (0.72, 1.73)		1.12 (0.71, 1.74)
**Fever**				
No		1		1
**Yes**				
Mild		1.34 (1.14, 1.58)		1.34 (1.14, 1.57)*
Moderate		0.99 (0.84, 1.16)		0.99 (0.83, 1.16)
Severe		0.90 (0.62, 1.28)		0.89 (0.62, 1.29)
Cut point 1	0.07 (0.06, 0.08)	0.34 (0.23, 0.49)	1.26 (0.88, 1.79)	0.33 (0.22, 0.50)
Cut point 2	1.04 (0.92, 1.076)	0.34 (0.23, 0.49)	4.25 (2.98, 6.06)	0.31 (0.20, 0.47)
Cut point 3	1.04 (0.59, 0.69)	4.73 (3.02, 17.47)	89.12 (61.54, 29.2)	4.65 (2.84 7.55)
SD	0.85 (0.77, 0.93)	0.48 (0.42, 0.53)	0.66 (0.59, 0.73)	0.42 (0.38, 0.47)
ICC (%)	18.01 (15.28, 20.8)	6.5 (5.09, 7.87)	11.69 (9.59, 13.94)	5.09 (4.21, 13.38)
PCV (%)	0	43.53	22.35	50.58
MOR	2.24 (2.08, 2.42)	1.58 (1.49, 1.70)	1.87 (1.75, 2.00)	1.49 (1.43, 1.56)
LOOIC	18,805.2	17,827.6	18,705.5	17,761.6

## Discussion

Childhood is a critical time for growth and development of a person and is a key stage in the establishment of their physical and mental abilities**.** Malnutrition including anemia during childhood had short-term and long-term effects on their growth and development^44^.

The aim of this study was to identify the determinants of anemia levels among children aged from 6 to 59 months based on Bayesian hierarchical statistical approach. In multivariable ordinal analysis child age, sex, maternal education, wealth index, stunting, history of fever, deworming, birth size, multiple birth, ANC visit, and maternal anemia were significantly associated with anemia level.

This study revealed that age of the child had different effect on their anemia level. The risk of anemia lowers when the children were getting older. This finding was supported by previous studies that states older children had less risk for anemia^11,45^. The possible explanation for this finding could be the fact that poor infant and young children feeding practice in Ethiopia particularly during the initiation of complementary feeding^46^. Moreover, female children hand lower risk of having higher level of anemia. This result was supported by another studies^46,47^. This could be explained by boys had a greater absolute longitudinal growth than girls in early stages of life^48^ and they require more macro and micro-nutrients intake like iron to balance their metabolic needs^49^.

In this study, maternal education was significantly related with the level of anemia. Attending primary education lowers the risk on their children having higher order of anemia. The result of the study was consistent with the result of various studies^45,50,51^. This could be educated mothers have better health, nutrition knowledge and child rearing practices than uneducated ones which contribute to improved child nutrition^52^.

This study also showed that wealth index was a significant and independent predictor of anemia level. Children from families having better wealth index had lower risk of having higher level of anemia. The result agreed with previous studies that showed a higher risk of anemia in people with low socioeconomic status^50^, and it could be the fact that children from low socio-economic classes are likely to be from poorly educated parents and often have financial constraints. And, their parents cannot afford good health services, or they might not have access to health services.

According to this study, children from mothers having ANC visit had lower risk of having higher order of anemia. This finding was in agreement with study done in Ghana^53^. The possible explanation for this finding might be there are different services provided for mothers visiting health institution for ANC to enhance positive pregnancy experience and ensuring that babies have the best possible start in life. This services include iron and folic acid supplementation, preventive anti-helminthic treatment, malaria prevention through provision of insecticide treated bed net, and health education on child feeding practices^54^.This services had direct and indirect effect on anemia among under five children.

Child size at birth and stunting were also significant and independent predictors of anemia level among children aged from 6 to 59 months. This finding were supported by other findings^55,56^. The possible reason could be since both anemia and stunting are a kind of malnutrition often share common causes. Besides, the gastrointestinal disturbance in malnourished individuals may reduce absorption essential micronutrients for erythropoiesis, and contribute towards development and worsening of anemia^16^.

This study also shows that fever had different effect on the levels of anemia. Children with history of fever had higher chance of having mild anemia. This finding were supported by other studies^57,58^. This might be related with children are the vulnerable groups for malaria infection and other febrile illness which result in anemia due to destruction of RBCs^59^.

Provision of drug for deworming of pre-school children’s and maternal anemia had significant effect on their anemia level. Unlike maternal anemia, provision of drugs for deworming had different effect on anemia level but it had lowered the risk of having moderate anemia. This finding agreed with different studies^60–64^. The possible reason for this association might be that both mothers and children had common dietary conditions. Moreover, maternal anemia could reduce the amount of iron secreted by the breast milk that might be insufficient for daily iron requirement of the child.

In addition to taking of drug for deworming, fever and child size at birth, children having twin had different effects on levels of anemia with children having twin had higher risk of having moderate level of anemia relative mild anemia. The possible reason for this result could be multiple pregnancy increases the risk of preterm birth and low birth weight. It also result in maternal anemia due to intra and post-partum bleeding^65^.

### Strength and limitation of the study

This main strength of this study was that it used national representative large datasets and the data were weighted to assure its representativeness. Besides, the data was analyzed using Bayesian statistical approach that includes additional information in addition to the data. Perhaps, this study uses non-informative priors which lowers weight given for prior information’s due to lack of sources. Besides, this study uses secondary data which was obtained from cross sectional survey. As a result, it does not allow to establish a cause-and-effect relationship. Moreover, this study did not include all modifiable risk factors like recent status/history of infectious disease particularly those which have a potential effect on erythropoiesis such as HIV and tuberculosis. In addition, the data were collected in the community level so there may be risk of recall bias.

## Conclusions

The prevalence of anemia among children aged 6–59 months anemia was found to be a severe public health problem. Children age, sex, maternal education, child stunting, history of fever, multiple birth, birth weight, deworming and maternal anemia were the most important determinants of anemia levels among children aged from 6 to 59 months. But the likelihoods of being severely anemic as compared to being moderately/mildly/non-anemic, of being severely/moderately anemic as compared to being mildly/non-anemic and of being severely/moderately/mildly anemic as compared to being non-anemic were not similar for child age, birth weight, history of fever, multiple birth and deworming of children keeping all other variables constant. Therefore, intervention efforts to control and prevent anemia in Ethiopia requires targeting of these hindering factors by taking special attention to mothers who had no formal education, poorest families, multiple births, LBW babies, stunted children, children with history of fever and maternal anemia.

## Data Availability

The datasets used and/or analyzed during the current study available from the corresponding author on reasonable request.

## Abbreviations

ANC: Antenatal care
CDI: Community Development Index
DHS: Demographic and Health Survey
EDHS: Ethiopian Demographic and Health Survey
Hgb: Hemoglobulin
MM: Mass media exposure
RBC: Red blood cells
Vit A: Vitamin A supplementation
WHO: World Health Organization
